# Distribution, Bioaccumulation, and Risks of Pharmaceutical Metabolites and Their Parents: A Case Study in an Yunliang River, Nanjing City

**DOI:** 10.3390/ijerph20042967

**Published:** 2023-02-08

**Authors:** Zhenhua Yan, Yixin Zhou, Yan Zhang, Xiadong Zhang

**Affiliations:** 1 Key Laboratory of Integrated Regulation and Resource Development on Shallow Lakes of Ministry of Education, Hohai University, Nanjing 210098, China; 2 College of Environment, Hohai University, Nanjing 210098, China; 3 Institute of Ocean and Offshore Engineering, Hohai University, Nantong 226018, China

**Keywords:** pharmaceutical, metabolite, accumulation, risk assessment, fish

## Abstract

The occurrence, bioaccumulation, and risks of 11 pairs of pharmaceutical metabolites and their respective parents were investigated in the water, sediment, and fish of an urban river in Nanjing city, China. The results showed that most of the target metabolites and their parents were detected in all water samples, with concentrations ranging from 0.1 ng/L to 72.9 ng/L. In some cases, the concentrations of metabolites in water were significantly higher than their parents, with fold changes reaching up 4.1 in the wet season and 6.6 in the dry season, while in sediment and fish, a lower concentration was observed in most cases. A lowered concentration of detected pharmaceuticals was observed in the dry season when compared to the wet season due to the seasonal variation in pharmaceutical consumption and overflow effluent. The bioaccumulation of pharmaceuticals in different fish tissues were detected with a descending order of overall concentration as gill > brain > muscle > gonad > intestine > liver > blood. In addition, the concentrations of both metabolites and their parents also decreased along the river in two seasons. However, the concentration rates of metabolites and their parents were significantly altered along the river in both water and sediment. The relatively high concentration proportions of the detected pharmaceuticals in water suggested that pharmaceuticals were more likely to apportion in water than in sediment, especially for the metabolites. Meanwhile, the rates of the metabolite/parent pairs between fish and water/sediment were generally lower, indicating the higher excretion capacity of metabolites from fish than their parents. Most of the detected pharmaceuticals had no impact on aquatic organisms. However, the presence of ibuprofen posed a medium risk to fish. Compared to the parents, metabolites showed a relatively low risk value but a high contribution to the total risk. It highlights that metabolites in the aquatic environments cannot be ignored.

## 1. Introduction

Pharmaceuticals play a key role in protecting public health and treating disease. Increasing with population growth and new pharmaceutical production, pharmaceuticals are hugely consumed by humans and animals, leading to a continuous entrance into the aquatic environment through different routes, such as direct discard, wastewater treatment plants, or livestock industry [1]. Since pharmaceuticals were originally designed to treat diseases and had bioactivity, a series of serious risks may be posed by these compounds to non-target organisms, thereby threatening human health and ecosystem security. For instance, the presence of antibiotics may promote the generation and spread of antibiotic resistance genes in the global lake [2]. The residuals of antidepressants in water disrupt the neurotransmitter transmission in the teleost, then impacting the growth and development of aquatic organisms at individual and population levels [3]. Hence, there is a growing concern about the occurrence and risk of pharmaceuticals in different water bodies. However, recent studies mainly focused on the pharmaceutical parents, and the threat from the metabolites was ignored [4].

After consumption in humans and biota, pharmaceuticals are rapidly excreted from the body via urine and feces as the parents or metabolites. There is no doubt that the parent pharmaceuticals are present worldwide in different aquatic environments. Nevertheless, only a few recent reports confirmed the presence of metabolites in different waters. For example, Petrie et al. [5] found that more than 20% of the newly recorded pollutants in UK waters were the products of metabolism. Zhou et al. [6] also summarized that approximately 66 pharmaceutical metabolites were recorded in European waters. In some cases, the concentrations of metabolites in water can reach levels similar to or even higher than the corresponding parents. In the coastal waters of Greece, the most common pharmaceutical was the metabolite desmethylvenlafaxine (OVM-VFS), while its parent, venlafaxine (VFS), was not detected [7]. A study conducted by de Oliveira et al. [8] also showed that metabolite concentration of gemcitabine was significantly higher than its parent in the effluent from a Brazilian hospital, with a high concentration of 116 μg/L. These findings suggest that the metabolites of pharmaceuticals are also widely present in waters, which may further interact with the environment and affect organisms.

The presence of the pharmaceutical metabolites in water may show the same modes of action on non-target organisms as their parents, leading to the same effect on organisms. After examining the aquatic toxicity of the antibiotic clarithromycin and its two metabolites, Baumann et al. [9] found the metabolite 14-hydroxy(R)-clarithromycin showed a similar toxic effect to its parent clarithromycin. Huang et al. [10] also suggested that the locomotor performances in zebrafish (*Danio rerio*) larvae were significantly affected by three metabolites, norfluoxetine (NFLX), OVM-VFS, and or sertraline (NDS), and this effect may be at a similar level to the corresponding parents. In addition, Arnnok et al. [11] showed a significant accumulation of NDS in the brains and livers of captured fish, which was much higher than the native sertraline (SER). The high accumulation potential of metabolites in organisms may mean high toxicity. In the work by Fong and Molnar [12], the metabolite NFLX can induce spawning in the freshwater bivalve zebra mussels (*Dreissena polymorpha*) and dark false mussels (*Mytilopsis leucophaeata*), which was 10 times higher than that induced by the parent fluoxetine (FLX). Taken together, the metabolites in water may be shown to be the same or even more toxic than their parents to organisms, resulting in a significant underestimation of the ecological risks based on the current assessment.

The Yangtze River, the third largest river in the world, has been seriously polluted by various compounds. After screening 1200 organic pollutants in Yangtze Estuary water, Li et al. [13] detected a total of 131 pollutants in the river, with total concentrations ranging from 1.8 to 9.7 μg/L. Some pollutants that occur at low concentrations may also pose high ecological risks. However, most existing studies mainly focused on the presence of pharmaceutical parents that occur in the Yangtze Estuary, such as antibiotics [14], estrogens [15], and several pharmaceuticals [16]; few studies have been conducted to characterize the occurrence of metabolites, especially in an urban river heavily influenced by human activity.

Therefore, the main aim of this study is to understand the occurrence and risks of different pharmaceutical metabolites and their corresponding parents in an urban river, since it has been recognized as the gathering place of pharmaceuticals in the urban region which is directly associated with human health. The temporal and spatial variation of pharmaceutical metabolites and their parents in the urban river were analyzed, including in surface water and sediment. The accumulative concentrations of these pharmaceuticals in native fish were also confirmed. The related risks to different riverine organisms were then assessed with the method of risk quotient.

## 2. Materials and Methods

### 2.1. Chemicals

A total of 11 pharmaceutical metabolites and 11 of their parents were selected as target pharmaceuticals in this study based on the occurrence of pharmaceuticals in the aquatic environment of the lower reach of the Yangtze River Delta Region [14,16], including sulfonamide antibiotics, nonsteroid anti-inflammatory drug, fibrate drug, and psychoactive drug. Given that a pharmaceutical has more than one metabolite, the bioactive one detected most frequently in water was used in this study. The basic physical and chemical properties of these pharmaceuticals are listed in Table 1. All of these pharmaceuticals were obtained from Dr. Ehrenstorfer (Augsburg, Germany) or J&K Chemical Ltd. (Shanghai, China) with a high purity grade (>98%). All solvents used in the study were purchased from Merck (Darmstadt, Germany) with a high-performance liquid chromatography (HPLC) grade.

### 2.2. Study Sites

**Figure 1:**
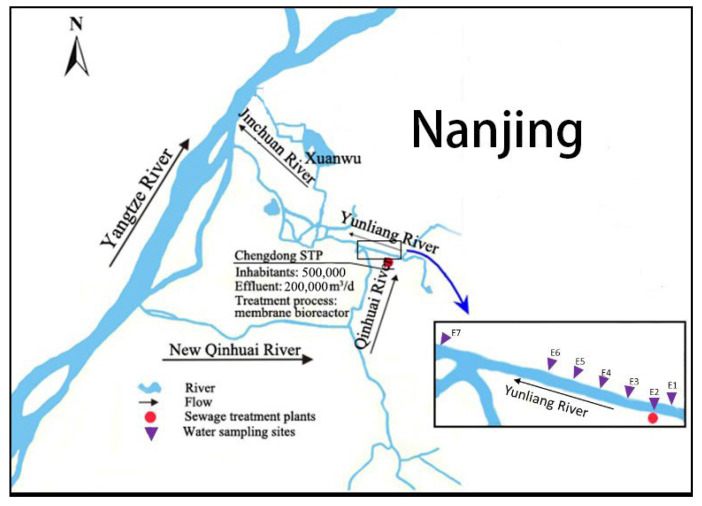
Sampling sites in the urban river.

The current study was conducted in an urban river called the Yunliang river affected by Chengdong sewage treatment plant (STP) effluents in Nanjing, China. Seven sampling sites were selected at the upstream (200 m), the outfall, and the downstream (200, 400, 600, 800, and 2000 m) of STP effluent, represented by the symbols E1-E7. A detailed map of the sampling sites is provided in Figure 1. The sampling campaign was conducted in July 2020 and January 2021 to represent the wet season and the dry season.

### 2.3. Sample Collection

At each site, both water and sediment samples were sampled with three replicates. Surface water was collected from 30 cm below the water surface using a 1.5 L stainless-steel water collector. Simultaneously, surface sediment was collected 5 cm from the top of the sediment with a stainless-steel grab sampler. All samples were stored in dry ice and immediately transported to the laboratory for further treatment within 24 h.

Fish samples were synchronously sampled through fishing, which was conducted ranging from 500 m to 1.5 km downstream of the STP outlet within 4 h. Thus, the sampling sites of fish could not be accurately marked in the river. Due to the lack of a sufficient number of fish of the same species, only the female white semiknife-carp (*Reganisalanx brachyrostralis*) was used for further treatment to maintain the unity of biological samples. Given the fish collected in the dry season were not enough, a total of 12 fish samples collected in the wet season were then used for further treatment with a body weight of 25–32 g and body length of 10–13 cm. The captured fish were anesthetized using MS-222, and then immediately transported to the laboratory in dry ice for pre-processing within 24 h.

### 2.4. Analytical Methods

The water samples were treated with the solid phase extraction (SPE) method according to previous studies with some modulations [14]. Briefly, the water sample was filtered through a 0.45 μm-glass fiber filter to remove suspended particulate matter. After modulating the pH to 3, a water sample (1 L) was extracted using HLB cartridge (500 mg, 6 cc, Waters, MA, USA), which was activated with 6 mL methanol and 6 mL ultrapure water before loading. Then the cartridge was washed with ultrapure water (2 × 6 mL) and dried for 30 min under vacuum. Finally, the cartridge was eluted with methanol (2 × 6 mL). The collected eluate was evaporated to near dryness under a nitrogen stream. The residue was then reconstituted with 1 mL of methanol containing internal standards for further analysis.

The sediment samples were extracted with the QuEChERS procedure described by Santos et al. [17]. After freeze-drying and sieving, 5 g of sediment was mixed with 10 mL of ultrapure water and vortexed for 30 s. After adding 15 mL of acetonitrile containing 2% ammonia, the mixture was vortexed for 30 s again. Then magnesium sulfate (4 g) and sodium chloride (1 g) were added, and the mixture was immediately shaken and vortexed for 1 min. The mixture was then centrifuged for 5 min at 10,000 rpm, and the acetonitrile layer was transferred to a centrifuge tube containing 150 mg C_18_ and 900 mg magnesium sulfate. After vortexing for 1 min, the mixture was centrifuged for 5 min at 10,000 rpm. After that, the supernatant was collected and evaporated to near dryness under a nitrogen stream. The residue was then reconstituted as the water sample treatment.

For the biological samples, the fish were sacrificed and their serum, brain, kidney, muscle, egg, and gill were collected and weighed. Four fish were gathered as one sample. The extraction and purification of these samples were based on methods described elsewhere [16,18]. After freeze-drying for 24 h, 0.5 g of fish tissues (brain, kidney, muscle, egg, and gill) were extracted using an ASE 350 pressurized liquid extraction system (Dionex, Sunnyvale, CA, USA) and purified with an Oasis HLB cartridges (6 mL, 200 mg; Waters, Milford, MA, USA). Due to the small weight, the brain, kidney, and egg samples from a total of 12 fish samples were combined for extraction. The serum samples (1 mL) were homogenized with 4 mL of acetonitrile for 5 min. After ultrasound for 10 min, the mixture was centrifuged at 13,000 rpm at 4 °C for 5 min. The supernatant was then collected and the residue was extracted three times. All collected supernatants were evaporated to near dryness and purified via the SPE method.

The detection of all target pharmaceuticals was performed on a Waters Acquity ultra-performance liquid chromatography coupled with a Waters Acquity Xevo TQ triple-quadrupole mass spectrometer (UPLC-MS/MS; Waters, USA). All samples were chromatographically separated on a BEH-C18 column (100 mm × 2.1 mm, 1.7 μm) with gradient elution. The tandem mass spectra were equipped with an electrospray ionization source operated in positive ion mode (ESI+) or negative ion mode (ESI-). The multiple reaction monitoring modes were applied for the detection and quantification of all pharmaceuticals. Detailed information on the instrumental analysis is described in the Supplementary Material.

### 2.5. Validation Method

The analytical methods were also validated. A procedural blank, a solvent blank, and a field blank were added in every batch of eight samples. No target pharmaceutical was detected in the blank samples. The recovery assays of each pharmaceutical were conducted with different spiked concentrations in different environments. The matrix effect in different mediums was also detected. The concentration of each pharmaceutical was adjusted using the internal standard method and reported as ng/L for water and ng/g (dry weight) for sediment and biota (ng/mL for serum). Specific information regarding the recovery and matrix effect are listed in the Supplementary Materials. The method detection limit (MDL) and method quantitation limit (MQL) for each pharmaceutical was defined as a signal-to-noise ratio of 3 and 10, respectively. The MDL and MQL were reported in the ranges of 0.02–0.26 ng/L and 0.07–0.90 ng/L in water, 0.03–0.40 ng/g and 0.10–1.25 ng/g in sediment, and 0.02–0.48 ng/g and 0.08–1.51 ng/g in fish (0.04–0.10 ng/mL and 0.15–0.40 ng/mL for serum), respectively.

### 2.6. Ecological Risk Assessment

According to the guidance of the European Chemicals Agency and Medicines Agency, the potential environmental implication of the detected pharmaceutical metabolites and their parents in water were evaluated using the risk quotient (RQ) approach, which was calculated based on the laboratory toxicity data. The formula is as follows:

(1) RQ=MEC/PNEC

where MEC is the measured environmental concentration in different seasons and PNEC is the predicted no effect concentration to different organisms in water. Due to the complexity of aquatic ecosystems, it is necessary to assess the risk of detected pharmaceuticals to aquatic organisms at different trophic levels, including algae, invertebrates, and fish [16]. The PNEC value is calculated as follows:

(2) $$\mathrm{PNEC}=\left({\mathrm{EC}}_{50},{\mathrm{LC}}_{50}\;\mathrm{or}\;\mathrm{NOEC}\right)/\mathrm{AF}$$

where EC_50_, LC_50_, and NOEC are the median effective, lethal, and no observed effect concentrations of each pharmaceutical to different organisms, respectively. Assessment factor (AF) refers to the safety factor. When LC_50_ or EC_50_ is available, the AF value was 1000. Once the NOEC is available for one, two, or three trophic levels, the AF value was 100, 50, or 10 [14]. The toxicity data used in this study were collected from the literature or ECOSAR and are listed in the Supplementary Material. The ecological risks of each pharmaceutical to different organisms were classified into four levels based on the RQ values: RQ < 0.01, no obvious risk; 0.01 ≤ RQ < 0.1, low risk; 0.1 ≤ RQ < 1, medium risk; RQ ≥ 1, high risk.

### 2.7. Statistical Analysis

All collected data were analyzed by SPSS 22 software (Chicago, IL, USA). All data are presented as the mean ± standard deviation (SD). After testing the homogeneity of variance with the Shapiro–Wilk normality test and Levene test, a one-way ANOVA was performed to determine the differences between the concentrations of pharmaceutical metabolites and their parents in the same season. The differences were regarded as significant at *p* < 0.05.

## 3. Results

### 3.1. Occurrence and Distribution in Water

**Figure 2:**
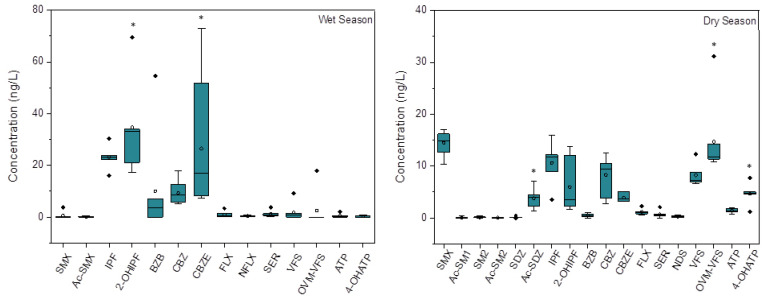
The concentrations of pharmaceutical metabolites and their parents in the water of Yunliang river in the wet and dry seasons. * refers to the significant difference between the concentrations of the metabolites and the related parents in the same season.

The average concentrations of the target pharmaceutical metabolites and their parents in water are shown in Figure 2. A total of 14 pharmaceuticals were detected in the wet season, including six metabolites and eight parents. In the dry season, a total of 18 pharmaceuticals were detected, and five of them were newly detected, including several sulfonamide metabolites and their parents. Among these pharmaceuticals, the metabolites of 2-hydroxy ibuprofen (2-OHIPF), Carbamazepine10,11-epoxide (CBZE), and the parent ibuprofen (IPF) and carbamazepine (CBZ) were always detected at high concentrations in the wet season, with the values ranging from 5.5 to 69.5, 7.5 to 72.9, lower than MQL to 30.3, and 5.1 to 17.8 ng/L, respectively. These broad concentrations suggest a heterogeneous distribution of pharmaceuticals in this river. The highest concentrations of these pharmaceuticals were generally higher than those of pharmaceuticals detected in the related Qinhuai River and the Yangtze River in China [14,16]. Given that the Yunliang river is a branch of the Qinhuai River and the Yangtze River, the more serious pollution in this river suggests the branch is an important source of pollutants in the main rivers. Compared to the wet season, the detected concentrations of 2-OHIPF, CBZE, and their parents IPF and CBZ in the dry season were much lower, with the highest concentrations of 13.70, 5.10, 15.95, and 12.60 ng/L, respectively. The seasonal variation of these pharmaceuticals in concentrations may be partly attributed to the seasonal variations in usage and consumption since the related diseases may be seasonal. In addition, the high concentrations detected in the wet season may also result from the frequent overflow due to heavy rainfall during this season. Similarly, Munro et al. [19] reported that the concentrations of caffeine, cocaine, and benzoylecgonine in the Thames Estuary of London could be elevated within 24 h by combined sewer overflow events due to the drastic resuspension in the wet season.

**Figure 3:**
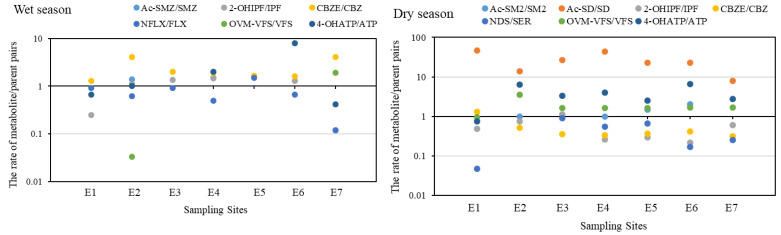
The concentration rates of metabolite/parent pairs in different sites in the water of Yunliang river in the wet and dry seasons.

To compare the concentrations of pharmaceutical metabolites and their parents in water, the concentration rates of selected pharmaceutical metabolite/parent pairs in different sites were calculated, and the results are shown in Figure 3. The metabolite concentrations were higher than their parents in most cases among these comparable pairs, with a concentration rate reaching up 4.1 in the wet season (except for Ac-SD/SD with rates from 8.0 to 47.0) and 6.6 in the dry season. Similar results have been reported by de Oliveira et al. [8], who found that the metabolite concentration of gemcitabine in the effluent of a Brazilian hospital reached up to 116 μg/L, significantly higher than its parent. Given that metabolites may be more toxic than their parent compounds in some cases [4], metabolites in water may pose a higher risk to aquatic organisms than their parents. These findings suggest that the residual metabolites in water could not be ignored due to their high concentrations and risks. Among these pharmaceutical metabolite/parent pairs, the concentrations of psychoactive metabolites were comparable to their parents in most cases. It suggests that psychoactive pharmaceuticals may be more resistant to degradation than the other ones. Similar results have been demonstrated by Svendsen et al. [20], who found that the degradation rate constant of psychoactive citalopram was much lower than that of ibuprofen by biofilm reactors. For the same metabolite/parent pairs, the concentration rates of Ac-SD/SD, OVM-VFS/VFS, and 4-OHATP/ATP in the dry season were higher than that in the wet season, whilst the 2-OHIPF/IPF and CBZE/CBZ showed a contrary result. This seasonal variation of the concentration rates may be partially caused by the changes in temperature or parent concentrations, which has been demonstrated by Lin et al. [21] and Svendsen et al. [20]. Taken together, more attention should be paid to the occurrences of the metabolites in the receiving water.

**Figure 4:**
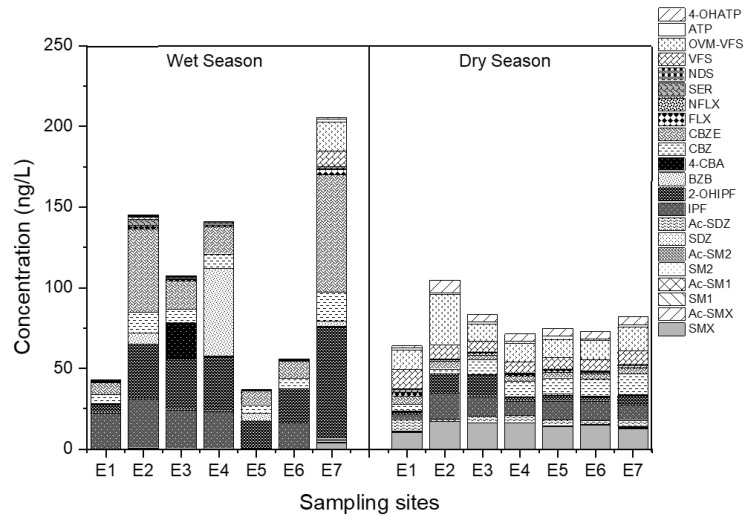
The spatial distribution of pharmaceutical metabolites and their parents in the water of the Yunliang river in different seasons.

The spatial distribution of the target pharmaceuticals is presented in Figure 4. There is no surprise with regard to the high concentrations of pharmaceuticals detected in site E2 in both wet and dry seasons due to the direct discharge of effluent from the Chengdong STP. In fact, STPs have been regarded as the main source of pharmaceutical pollutants in urban rivers due to the inefficient degradation and removal of pharmaceuticals by traditional biochemical treatment. As reported by Gros et al. [22], the removal efficiencies of antibiotics and β-agonists in the STP in Spain generally ranged from 40 to 60%, or in some cases no elimination, which led to the frequent detection of these pharmaceuticals in the receiving river. In addition, Liu et al. [23] found that the state-of-the-art treatment processes in STPs in China were generally inefficient in removing pharmaceuticals. Only 14% of pharmaceuticals could be removed effectively, with a removal efficiency of more than 70%, while more than 51% of them had a removal rate below 30%. In the downstream, the detected concentrations of pharmaceuticals decreased along the river. The decreased concentrations may be attributed to the dilution effect or adsorption on the particulate matter and colloid in water [24], which would further settle into the sediment along the river. In addition, a previous study suggested that the abiotic or biotic processes in rivers, such as photolysis or microbial degradation, could also enhance the concentrations of metabolites downstream from parent degradation [25]. Hence, the concentration rates of pharmaceutical metabolite/parent pairs were further compared along the river. It shows that the rate increased at sample site E2 in most cases, suggesting that the presence of metabolites near STPs may be mainly discharged from the effluent of the Chengdong STP; whilst in the lower reaches of this river, the concentration rates were not significantly altered. This means that both the metabolites and their parents were removed simultaneously from the abiotic or biotic processes in the lower reaches of the river, thereby leading to a decrease in the total concentrations. Moreover, there is no significant change in the variation tendency of the rate of metabolite/parent in the wet and dry seasons along the Yunliang river. In addition, the highest concentration of pharmaceuticals was observed in sampling site E7, which is located at the confluence of the Yunliang and New Qinhuai rivers. Due to the violent turbulent mixing in this confluence zone, high concentrations of pharmaceuticals may be released from the sediment through resuspension [26].

### 3.2. Occurrence and Distribution in Sediment

**Figure 5:**
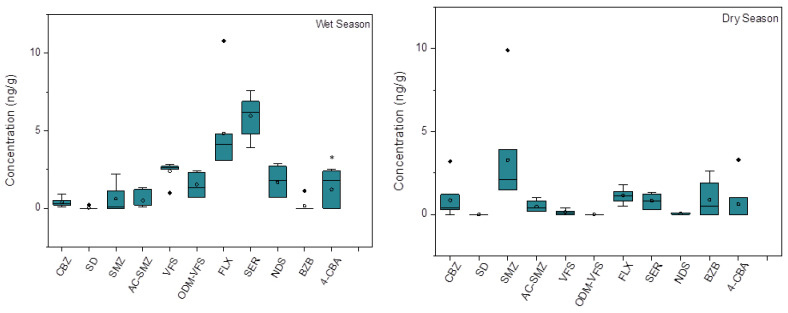
The concentrations of pharmaceutical metabolites and their parents in the sediment of Yunliang river in the wet and dry seasons. * refers to the significant difference between the concentrations of metabolites and their related parents in the same season.

The concentrations of different pharmaceuticals in the sediment are shown in Figure 5. A total of 11 pharmaceuticals were detected in the wet and dry seasons, including four metabolites and seven parents. Among these pharmaceuticals, the antidepressants FLX and SER were always detected at high concentrations in the wet season, with a concentration range from 3.1 to 10.8 ng/g and 3.9 to 7.6 ng/g, respectively. In the dry season, these antidepressants were also 100% detected with a low concentration ranging from 0.5 to 1.8 ng/g and 0.3 to 1.3 ng/g, respectively. Compared with the highest concentrations of these pharmaceuticals detected in the sediment of rivers across Nanjing and in the Yangtze River [14,16], the concentrations observed in this study were comparatively high, which is consistent with that detected in the water. Similar to the water, the concentrations of pharmaceuticals in the sediment in the wet season were comparatively higher than that in the dry season in most cases, especially for the antidepressants FLX, SER, and VFS. The consistency in the seasonal variation of pharmaceuticals in sediment and water suggests that the residues of pharmaceuticals in sediment may mainly come from sedimentation. As the commonly used antidepressants in clinics, the wide detection of FLX, SER, and VFS in receiving rivers implies an increasing number of people who may be suffering from depression due to increasingly competitive pressures, especially due to COVID-19 [27]. Meanwhile, the metabolite of SER, called NDS, was also detected in most sites in the sediment, while it has not been detected in any sites in water. This metabolite is easy to be partitioned into the sediment due to the high lipophilicity.

**Figure 6:**
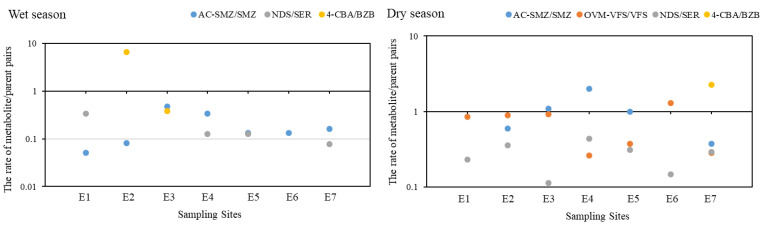
The concentration rates of metabolite/parent pairs in different sites in the sediment of Yunliang river in the wet and dry seasons.

Different from the water phase, the low concentration rates of pharmaceutical metabolite/parent pairs in sediment showed that the concentrations of the pharmaceutical metabolites in sediment were lower than their parents in sediment (Figure 6). This result may be caused by the high concentrations of dissolved organic matter in the sediment, which could result in a high degradation rate of metabolite in the environment [28]. In addition, for the same metabolite/parent pairs, the concentration rates of Ac-SMZ/SMZ and OVM-VFS/VFS in the dry season were generally higher than that in the wet season, which was similar to their seasonal variation in water. This result once again confirmed that the pharmaceuticals in sediment may come from the water phase.

**Figure 7:**
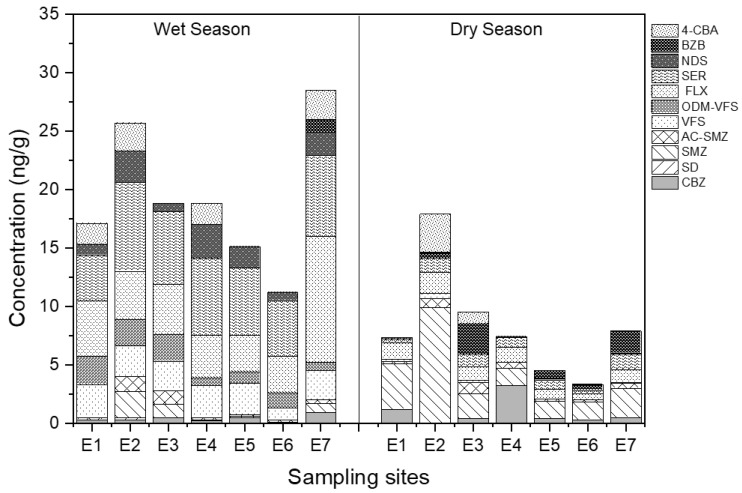
The spatial distribution of pharmaceutical metabolites and their parents in the sediment of the Yunliang river in different seasons.

The spatial distribution of the detected pharmaceuticals in sediment is shown in Figure 7. Similar to the water, high concentrations of pharmaceutical metabolites and their parents are also observed in the sediment located near site E2 in two seasons. Subsequently, the total concentrations decreased along the river due to the removal of the abiotic and biotic factors. However, in the confluence of E7, the total concentrations were reversely enhanced to 28.5 ng/g in the wet season and 7.9 ng/g in the dry season, which may be attributed to import from a tributary in the water. The total concentrations in the wet season were much higher than that in the dry season in sediment, which also corresponded with that in the water. Among these pharmaceuticals, a lower concentration was observed with sulfamethoxazole (SMX), which is always detected at a high level in the water. It implies that SMX may be preferentially present in the water phase. The low octanol–water distribution coefficient (0.89) suggests that the SMX has a strong hydrophilicity and is not easily absorbed by sediment [29]. On the contrary, the antidepressants and their metabolites, including FLX, SER, NDS, VFS, and OVM-VFS, were frequently detected in the sediments with a high proportion, which may be attributed to their relatively high octanol–water distribution coefficient. For the concentration rates of pharmaceutical metabolite/parent pairs in sediment, their values were not significantly altered along the river in the wet and dry seasons (Figure 6), indicating a simultaneous and similar removal of metabolites and their parents in the sediment.

### 3.3. Bioaccumulation in Fish

**Figure 8:**
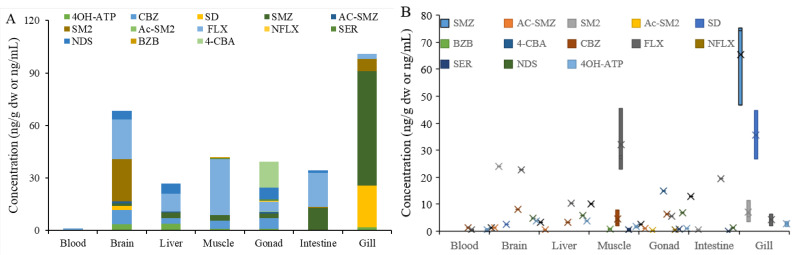
Accumulation of pharmaceutical metabolites and their parents in different fish tissues, (**A**) total concentration, and (**B**) concentration distribution.

The accumulative concentrations of pharmaceutical metabolites and their parents in different fish tissues are presented in Figure 8. A total of 13 pharmaceuticals were detected in the collected fish, including six metabolites and seven parents. In the same tissue, the detected rates of these pharmaceuticals ranged from 29% in the blood to 77% in the spawn. Compared to the total concentrations of pharmaceuticals in five tissues (blood, brain, liver, muscle, gonad, intestine, and gill), the gill had the highest concentration (100.80 ng/g) while the intestine had a lower concentration (34.30 ng/g d.w.). Among the detected pharmaceuticals in fish, the highest concentration was 75.30 ng/g of SMZ in the gill, which was generally higher than that detected in the fish collected from the related Qinhuai River [30]. Given that waterborne pollutants could be taken up by fish through the gill and intestine, the different concentrations in two tissues suggest that female white semiknife-carp may mainly take up these pharmaceuticals from the gill but not the intestine, especially for the sulfonamides. In general, the gill is the first organ to be in contact with water, so it can be the relevant site of interaction with pollutants [31]. Liu et al. [32] have found that the antibiotic erythromycin concentrations in the gill of crucian carp (*Carassius auratus*) tended to be the highest among all the tested tissues. Cheng et al. [33] also confirmed a preferential accumulation in the gill of carp by dibromodiphenyl ether.

A high concentration of pharmaceuticals was also detected in the brain, and FLX contributed more than 30%. The blood–brain barrier plays an important role in protecting the brain from pharmaceuticals, and most pharmaceuticals cannot enter the brain from the blood. The high levels of FLX residue suggest that the brain is the targeted tissue of FLX in wild fish. As one of the most popular antidepressants, the residual FLX in the brain may further inhibit the reuptake of serotonin from cell membranes, leading to different neurotoxicity in fish, including the behavioral alterations of anxiety, aggression, and compulsivity [34]. It should also be noted that the highest detection frequency of pharmaceuticals was in gonad tissue. The presence of nine pharmaceuticals in the gonadal indicates that these pharmaceuticals could cross the blood–ovary barrier and increase the risk of transgenerational toxicity. Zhou et al. [35] found that the distribution of ethylhexyl salicylate in gonads could be transferred to offspring through reproduction, thus influencing its growth, development, and locomotor vitality. In addition, the muscle also had a high concentration of pharmaceuticals. Similar results have been reported by Liu et al. [32] and Belfroid et al. [36], who found a generally higher accumulation of erythromycin and bisphenol A in the muscle of fish than that in the other tissues. Meanwhile, the high residue of pharmaceuticals in muscle, especially for FLX, may elevate their risks to human health through food chain transformation.

Compared to the gill and brain, the liver had a lower pharmaceutical concentration. The liver is the main metabolic tissue in fish, which could quickly metabolize and eliminate the accumulated pharmaceuticals to reduce the harm of these pharmaceuticals to organisms [34]. Hence, the low concentration in the liver in this study might indicate that the liver in wild fish has sufficient capacity to metabolize and excrete these pharmaceuticals from the body. These pharmaceuticals will be further transported through the blood and be excreted from the body [35]. So it is not surprising to find the lowest concentrations of pharmaceuticals in the blood.

Relative to the parent pharmaceuticals in fish, the metabolites were mostly detected with low concentrations, except for the metabolite of 4OH-ATP and NDS, which had a high concentration reaching up 3.85 and 5.77 ng/g, respectively. For the 4OH-ATP, its parent ATP was not detected in any fish samples. Given the 4OH-ATP was also detected in water, the accumulated 4OH-ATP in fish may originate from the water or the metabolism of its parent ATP in the body. In addition, the metabolite of NDS was detected in most tissues in the fish, while it did not present in the water. It implies that the accumulated metabolite NDS in fish may come from the metabolism of its parent in the body, especially in the liver. The high bioaccumulation of metabolites in aquatic organisms was also confirmed in field studies. For instance, after analyzing the tissue distribution of different antidepressants from 11 species of fish inhabiting the Niagara River, Arnnok, et al. [11] found a significant accumulation of NDS in the brain and liver of fish, while no significant bioaccumulation of the parent pharmaceutical SER was observed in any fish. Brooks et al. [37] also found that the metabolite NDS in wild fish reached higher concentrations than its parent. Hence, the accumulation of the pharmaceutical metabolite in fish could not be ignored. In addition, the concentration rates of pharmaceutical metabolites and their parents in fish were calculated. Due to the lack of the corresponding metabolite and parent in the same fish tissues in most cases, only the Ac-SMZ/SMZ and NFLX/FLX were calculated, with a value of less than one in all cases.

### 3.4. Environmental Implication

**Figure 9:**
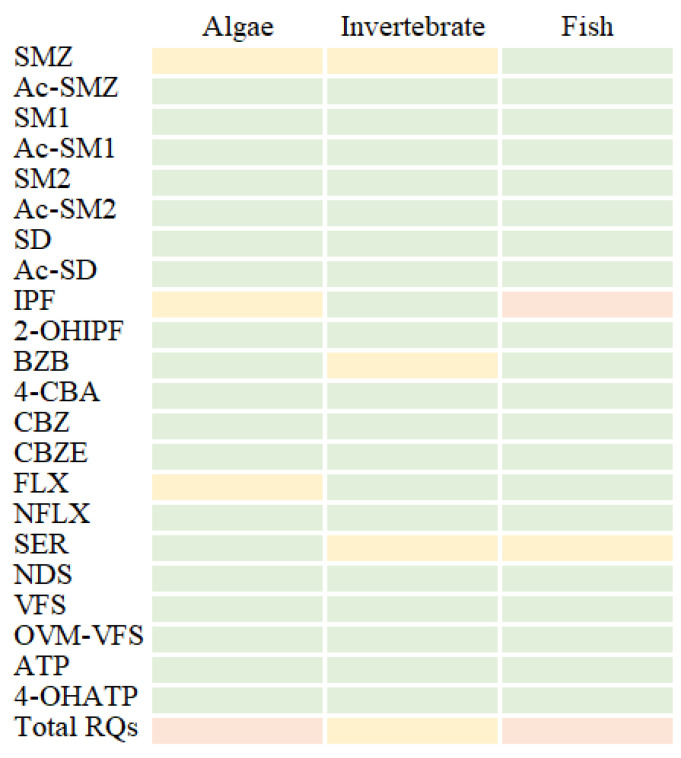
The risks of pharmaceutical metabolites and their parents to algae, invertebrates, and fish.

The average risks of different pharmaceuticals and their metabolites to different trophic levels of aquatic organisms (algae, invertebrates, and fish) and the supporting information are shown in Figure 9.

Among these pharmaceuticals, average RQs of SMX to algae and invertebrates were higher than 0.01, indicating a low risk. Similar results have been also reported in a previous study, in which the sulfonamide antibiotics always had a comparatively high risk to algae in different rivers [38]. Compared with algae, the risks induced by sulfonamides to fish were relatively low, suggesting that the sulfonamides may cause little harm to fish in urban rivers. Lu et al. also confirmed that fish could almost not be affected by antibiotics in Laizhou Bay [39]. The anti-inflammatory pharmaceutical IPF, quite to the contrary of sulfonamides, always posed a medium risk to fish. In addition, exposure to IPF also resulted in a low risk to algae, while the invertebrates were not affected. Previous studies have also shown that IPF posed a high ecotoxicological risk to different aquatic organisms [40]. Considering the high RQ values and the common fates of IPF in water, the urban river ecosystems may be threatened by IPF residues, especially for fish. Moreover, the presence of antidepressant SER also showed a low risk to invertebrates and fish, as well as a low risk of FLX to algae and BZB to invertebrates. To characterize the worst-case scenario at each site, the RQ of each pharmaceutical and the related metabolite was also summed. The results showed that these pharmaceuticals posed a medium risk to algae and fish, and a low risk to invertebrates in total. Among these aquatic organisms, sulfonamides always posed a higher risk to algae than invertebrates and fish. On the contrary, psychoactive pharmaceuticals generally posed a higher risk to invertebrates and fish than to algae, which may be related to their different toxicity mechanisms in organisms. In addition, the weak relationship between the RQs and the accumulative concentrations of different pharmaceuticals suggests that the risks of pharmaceuticals may be not only caused by their accumulation but also mostly induced by their toxicity effects and the related mechanisms.

Compared with the parent pharmaceuticals, the metabolites always posed a significantly lower risk to all organisms, with a contribution rate of 0.04–7.33% to fish, 1.42–18.05% to invertebrates, and 1.01–16.57% to algae, respectively. Even if the concentrations of metabolites were higher than their parents in water, the related RQs were still at low levels. However, the presence of the metabolites may further enhance the risks caused by their parents, in particular with the metabolites playing the same mode of action in organisms as their parents, which could lead to the same or even more toxic levels than their parents. This finding suggests that the significant risks caused by pharmaceutical metabolites in some cases could not be ignored. Since pharmaceuticals in water may interact with each other at a physicochemical level or through their toxicokinetics and toxicodynamics [41], the cumulative risks used in this study may underestimate or overestimate the total risks. Thus, more attention should be paid to their interaction with aquatic organisms, especially to the co-existence of the parent pharmaceuticals and their metabolites.

### 3.5. Proportions of Pharmaceuticals in Different Matrices

**Figure 10:**
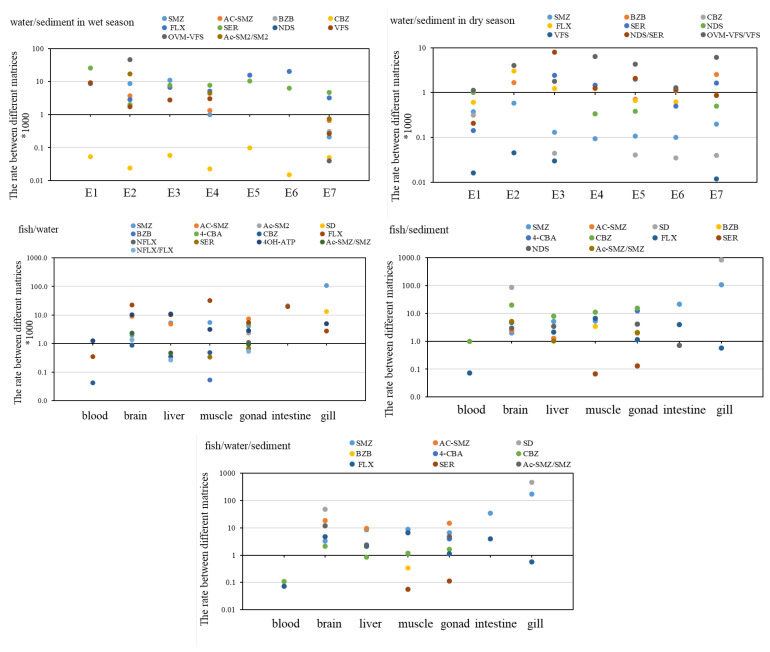
The concentration proportions of different pharmaceuticals between different matrices.

To obtain the concentration proportions of target pharmaceuticals between different matrices, the rates of the corresponding pharmaceuticals and the metabolite/parent pairs in different matrices were calculated, including water/sediment, tissue/water, tissue/sediment, and tissue/water/sediment, and the results are showed in Figure 10.

For the corresponding pharmaceuticals, a higher concentration rate was observed in the water than that in the sediment in the wet season in most cases. However, in the dry season, the proportions of most pharmaceuticals in water were significantly lowered. This seasonal variation in the proportions may be caused by the heavy rainfall and hydraulic scour in the wet season, which could further enhance the resuspension of sediment and elevate pharmaceutical concentrations in water, as reported by Munro et al. [19]. Along the river, the rate of target pharmaceuticals in water gradually declined in two seasons, which may be attributed to the free settling of pharmaceuticals in water phase. In addition, a high concentration proportion was also observed between different fish tissues and water/sediment, suggesting that most pharmaceuticals tended to apportion in fish tissues from water or sediment, especially in the gill and the brain. Compared to sediment, the rates between fish tissues and water were much higher, with three orders of magnitude, indicating that the accumulation of pharmaceuticals in fish may mainly come from water bodies.

To obtain the concentration proportions of the metabolite/parent pairs, only the same pairs in different matrices were used for calculation. The rates of the corresponding pairs in water were much higher than that in sediment in the dry season, which further increased in the wet season. This finding means the metabolites may be more easily distributed in the water phase. Given that pharmaceuticals are mainly metabolized by the liver and excreted as hydrolysates in organisms, there is no doubt about the solubility of the metabolites in the water versus their parents. In addition, the high temperature and heavy rainfall in the wet season could also enhance the production of metabolites and the release from sediment [20,21]. Moreover, the rates of metabolite/parent pairs in water gradually also declined along the river in two seasons, indicating a free settling of both metabolites and their parents in water. For the proportions of the metabolite/parent pairs between fish tissues and water/sediment, a relatively lower rate was observed in fish than that of the corresponding pharmaceuticals, suggesting a higher excretion capacity of metabolite from fish than their parents.

## 4. Conclusions

This study revealed a new insight into the distribution, bioaccumulation, and risks of pharmaceutical metabolites and their parents in an urban river of Nanjing city. A heterogeneous distribution of pharmaceuticals was observed in the water, sediment, and fish, with a high concentration reaching up 72.9 ng/L, 28.5 ng/g, and 75.30 ng/g, respectively. The relatively high residual levels in water during the wet season may be partly attributed to the seasonal variations in pharmaceutical consumption and resuspension caused by heavy rainfall during this season. The metabolite concentrations in water were generally higher than their parents in most cases. The seasonal variation in the concentration rates between metabolites and parents may be partially caused by the changes in temperature and parent concentrations. On the contrary, a lower concentration rate of metabolite/parent pairs was observed in sediment and fish, highlighting a high removal of metabolites in these matrices. The concentration proportions between different matrices indicated that the pharmaceuticals were more likely to apportion in water than in sediment, especially for metabolites. The relatively low rate of metabolite/parent pairs between fish and water/sediment also indicated a higher excretion capacity of metabolites from fish than their parents. Among the detected pharmaceuticals in water, most of them posed no risk to aquatic organisms. Compared to the parents, the metabolites always posed a lower risk. However, the relatively high contribution to the total risk suggested that metabolites in water could not be ignored. Therefore, it is necessary to investigate the presence and related risks of pharmaceutical metabolites in the aquatic environment.

## Figures and Tables

**Table 1:** The target pharmaceutical metabolites and their parents used in this study.

Categories	Pharmaceutical Metabolites/Parents	Abbreviation	CAS	Molecular Weight	Structure Formula
Sulfonamide drug	Sulfamethoxazole	SMX	723-46-6	253.28	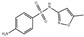
	N4-Acetyl sulfamethoxazole *	Ac-SMX	21312-10-7	295.32	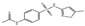
	Sulfamerazine	SM1	127-79-7	264.3	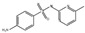
	Acetyl sulfamerazine *	Ac-SM1	127-73-1	306.34	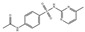
	Sulfadimidine	SM2	57-68-1	278.32	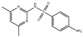
	Acetyl sulfadimidine *	Ac-SM2	100-90-3	320.37	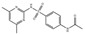
	Sulfadiazine	SD	68-35-9	250.28	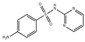
	Acetyl sulfadiazine *	Ac-SD	127-74-2	292.31	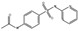
Nonsteroidal anti-inflammatory drug	Ibuprofen	IPF	15687-27-1	206.28	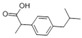
	2-Hydroxy ibuprofen *	2-OHIPF	51146-55-5	222.28	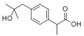
	Antipyrine	ATP	60-80-0	188.23	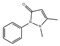
	4-Hydroxyantipyrine *	4-OHATP	1672-63-5	204.23	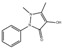
Fibrate drug	Bezafibrate	BZB	41859-67-0	361.82	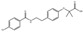
	4-Chlorobenzoic acid *	4-CBA	74-11-3	156.57	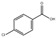
Psychoactive drug	Carbamazepine	CBZ	298-46-4	236.27	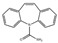
	Carbamazepine-10,11-epoxide *	CBZE	36507-30-9	252.27	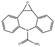
	Fluoxetine	FLX	56296-78-7	345.79	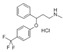
	Norfluoxetine *	NFLX	83891-03-6	331.76	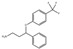
	Sertraline	SER	79617-96-2	342.69	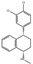
	Norsetraline *	NDS	91797-57-8	328.66	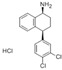
	Venlafaxine	VFS	99300-78-4	313.86	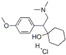
	O-Desmethylvenlafaxine *	OVM-VFS	93413-62-8	263.38	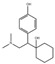

* Compounds are pharmaceutical metabolites.

## Data Availability

The data presented in this study are available at this article and Supplementary Materials.

## Supplementary Materials

The following supporting information can be downloaded at https://www.mdpi.com/article/10.3390/ijerph20042967/s1, Text S1: Details of instrumental analysis; Table S1: The gradient program of mobile phase in different modes; Table S2: Experimental conditions used for LC-MS/MS; Table S3: Recoveries of target pharmaceuticals and matrix effect in water, sediment and fish; Table S4: Aquatic toxicity data and the calculated PNEC values (ng/L) for algae, in-vertebrate and fish for target pharmaceuticals; Figure S1: The risks of pharmaceutical metabolites and their parents to algae (A), in-vertebrates (B), and fish (C) in different sampling sites. References [42,43,44,45,46,47,48,49,50,51,52,53,54,55,56] are cited in Supplementary Materials.
