# Supplementary material for: Distribution, Bioaccumulation, and Risks of Pharmaceutical Metabolites and Their Parents: A Case Study in an Yunliang River, Nanjing City
Source: Int J Environ Res Public Health. 2023 Feb 8;20(4):2967. doi: 10.3390/ijerph20042967 (PMC9964203; doi:10.3390/ijerph20042967)
Supplement: Supplementary file 1 [file ijerph-20-02967-s001.zip › ijerph-2115625-supplementary.pdf]

Supplementary Materials

Distribution, Bioaccumulation, and Risks of Pharmaceutical Metabolites and their Parents: a case study in the Yunliang River, Nanjing city

Zhenhua Yan [1,2,*], Yixin Zhou [1], Yan Zhang [1] and Xiadong Zhang [1,3]

[1] Key Laboratory of Integrated Regulation and Resource Development on Shallow Lakes of Ministry of Education, Hohai University, Nanjing 210098, China
[2] College of Environment, Hohai University, Nanjing 210098, China
[3] Institute of Ocean and Offshore Engineering, Hohai University, Nantong 226018, China
* Correspondence: hwahuer@hhu.edu.cn or hwahuer@126.com

This file includes:

Text S1. Details of instrumental analysis

Table S1. The gradient program of mobile phase in different modes.

Table S2. Experimental conditions used for LC-MS/MS.

Table S3. Recoveries of target pharmaceuticals and matrix effect in water, sediment and fish.

Table S4. Aquatic toxicity data and the calculated PNEC values (ng/L) for algae, invertebrate and fish for target pharmaceuticals.

Figure S1. The risks of pharmaceutical metabolites and their parents to algae (A), invertebrates (B), and fish (C) in different sampling sites.

Text S1. Details of instrumental analysis

All target pharmaceuticals were analyzed by a Waters Acquity UPLC-MS/MS system with an electrospray ionization source (ESI) in both positive and negative ionization modes. An Acquity UPLC BEH-C18 column (100 mm×1.7 μm×2.1 mm; Waters, USA) was used and maintained at 40 °C. In positive ionization mode (ESI+), the mobile phase consisted of eluent A (98% ultrapure water with 2% methanol containing 0.05% formic acid, v/v) and eluent B (acetonitrile). In negative ionization mode (ESI-), the mobile phase consisted of eluent A (98% ultrapure water 2% methanol containing 5 mM ammonium acetate) and eluent B (acetonitrile). The flow rate was set at 0.4 mL/min. The injection volume was 5 μL. The mobile phase gradient was described in Table S1.

Multiple responses monitoring (MRM) mode was used to identify and quantify target pharmaceuticals. The temperature of ion source was kept at 150 °C. High purity nitrogen and high purity argon were used as atomizing gas, desolvent gas and collision gas, respectively. The flow rates were 891 L/Hr and 0.15 mL/min, respectively. The capillary voltage was 2.0 kV, the temperature of desolvation was kept at 500 °C, and the flow rate of cone hole blowback air was kept at 50 L/Hr. The mass spectrometry conditions of target pharmaceuticals are shown in Table S2.

Table S1. The gradient program of mobile phase in different modes.

Mode	Time (min)	Mobile phase (%)	
		A	B
ESI+	0	90	10
	0.25	90	10
	3	10	90
	4	10	90
	4.01	90	10
	5	90	10
		A	B
ESI-	0	90	10
	2.5	90	10
	4	10	90
	5	10	90
	5.01	90	10
	6	90	10

Table S2. Experimental conditions used for LC-MS/MS

Target pharmaceuticals	Retention time, min	Precursor Ions, m/z	Production Ions, m/z	Cone Voltage, V	Collision Energy, eV	Modes
SMX	2.30	254	92	25	26	ESI+
Ac-SMX	3.08	296	134	12	6	ESI+
SM1	1.96	265	92	26	28	ESI+
Ac-SM1	1.81	307	172	30	20	ESI+
SM2	2.03	279	92	30	28	ESI+
Ac-SM2	1.91	321	134	40	26	ESI+
SD	1.57	251	92	25	27	ESI+
Ac-SD	1.71	293	65	50	34	ESI+
IPF	3.97	205	161	16	7	ESI-
2-OHIPF	2.59	221	177	16	7	ESI-
BZB	3.33	360	154	32	30	ESI-
4-CBA	2.95	155	111	24	10	ESI-
CBZ	2.82	237	165	30	42	ESI+
CBZE	2.45	253	180	28	32	ESI+
FLX	3.17	310	44	6	10	ESI+
NFLX	3.09	296	134	48	6	ESI+
SER	3.25	306	159	20	26	ESI+
NDS	3.17	292	159	8	24	ESI+
VFS	2.40	315	44	26	10	ESI+
OVM-VFS	1.93	264	58	12	16	ESI+
ATP	1.99	189	56	40	28	ESI+
4-OHATP	2.20	205	56	30	16	ESI+

Table S3. Recoveries of target pharmaceuticals and matrix effect in water, sediment and fish

Target pharmaceuticals	Recoveries (%)								
	Spiked in water		Matrix effect in water	Spiked in sediment		Matrix effect in sediment	Spiked in fish		Matrix effect in fish
	10 ng/L	100 ng/L		10 ng/g	100 ng/g		20 ng/g	100 ng/g	
SMX	90.4±5.5	93.0±3.0	81-92%	98.5±11.1	90.5±3.2	84-108%	75.0±3.4	84.1±3.4	81-96%
Ac-SMX	84.1±2.4	86.0±3.5	89-101%	109.4±7.4	113.1±5.4	95-111%	82.5±5.3	89.3±4.5	84-103%
SM1	76.1±4.1	81.0±2.0	83-107%	98.5±7.7	101.2±12.3	102-116%	89.3±3.2	111.1±2.8	89-101%
Ac-SM1	87.1±1.8	95.0±5.9	92-96%	88.9±7.5	74.5±6.4	79-96%	90.0±3.0	93.5±6.1	105-117%
SM2	92.9±3.9	97.0±5.5	78-91%	78.9±8.2	94.8±2.7	85-105%	75.6±9.7	78.0±6.8	84-108%
Ac-SM2	83.5±3.9	89.5±5.5	85-96%	90.8±4.3	93.4±5.4	91-114%	78.5±4.9	80.5±6.4	96-112%
SDZ	100.2±5.6	97.3±2.4	87-95%	73.8±6.8	86.7±14.4	96-105%	76.0±2.1	85.4±4.6	79-101%
Ac-SDZ	73.1±1.9	80.5±3.4	96-112%	79.5±10.4	97.2±5.2	84-103%	80.5±3.1	79.3±2.5	86-104%
IPF	101.0±6.8	110.1±2.1	79-101%	81.4±10.2	105.1±11.4	89-101%	70.0±6.8	74.4±7.6	92-99%
2-OHIPF	107.5±3.9	111.1±4.3	86-104%	108.1±7.6	92.7±7.5	105-117%	80.1±14.1	73.2±12.7	77-91%
BZB	106.3±4.7	99.9±0.6	92-99%	80.5±9.2	110.2±7.0	82-107%	90.0±4.1	86.1±9.2	85-101%
4-CBA	103.6±2.2	101.3±5.9	77-91%	86.9±8.7	103.4±7.7	96-104%	76.0±11.3	82.9±7.2	89-98%
CBZ	106.1±1.9	105.0±8.6	85-101%	93.4±4.3	92.7±9.4	87-112%	72.5±6.4	78.5±4.9	86-103%
CBZE	98.4±0.3	102.4±3.6	89-98%	74.4±7.5	85.4±14.5	98-111%	89.1±4.3	83.3±12	105-117%
FLX	89.7±3.9	88.1±7.2	96-108%	98.5±8.3	72.7±6.7	86-103%	108.0±5.3	113.1±6.3	95-116%
NFLX	75.5±8.0	84.0±9.3	83-105%	86.8±1.7	110.5±9.0	105-117%	107.0±2.9	119.4±7.6	97-103%
SER	93.5±11.4	101.4±6.4	98-113%	93.4±12.1	92.3±5.9	87-105%	89.0±4.2	83.8±2.6	96-106%
NDS	78.6±5.3	82.1±0.9	88-107%	79.8±4.6	108.5±7.2	84-111%	76.0±2.8	81.0±8.2	82-99%
VFS	88.9±3.9	118.0±3.3	79-111%	84.8±6.5	100.9±4.3	92-99%	81.9±3.2	87.1±3.5	79-101%
OVM-VFS	81.1±4.8	78.4±6.5	99-107%	98.7±11	104.6±2.7	97-118%	82.4±3.6	89.2±6.5	86-104%
ATP	94.0±2.5	97.1±5.3	102-116%	109.8±7.1	108.4±4.9	81-108%	75.0±6.9	80.5±4.8	92-99%
4-OHATP	92.1±3.6	96.3±6.5	92-99%	98.7±6.7	82.7±5.3	96-107%	85.1±6.9	82.4±4.8	77-91%

Table S4. Aquatic toxicity data and the calculated PNEC values (ng/L) for algae, invertebrate and fish for target pharmaceuticals.

Pharmaceuticals	Aquatic organisms	LC_{50}/EC_{50} (mg/L)	AF	PNEC (ng/L)	Reference
	algae	0.027	1000	27	[1]
SMX	invertebrates	0.21	1000	210	[2]
	fish	562.5	1000	562500	[3]
	algae	8.620	1000	8620	ECOSAR
Ac-SMX	invertebrates	515.820	1000	515820	ECOSAR
	fish	325.206	1000	325206	ECOSAR
	algae	1*	50	20000	[4]
SM1	invertebrates	1.563*	50	31260	[5]
	fish	56.2	1000	56200	[6]
	algae	13.242	1000	13242	ECOSAR
Ac-SM1	invertebrates	954.002	1000	954002	ECOSAR
	fish	530.033	1000	530033	ECOSAR
	algae	6.259	1000	6259	ECOSAR
SM2	invertebrates	2.045	1000	2045	ECOSAR
	fish	291.394	1000	291394	ECOSAR
	algae	6.329	1000	6329	ECOSAR
Ac-SM2	invertebrates	314.851	1000	314851	ECOSAR
	fish	255.099	1000	255099	ECOSAR
	algae	0.11	1000	110	[7]
SDZ	invertebrates	57	1000	57000	[7]
	fish	25900	1000	25900000	[8]
	algae	27.650	1000	27650	ECOSAR
Ac-SDZ	invertebrates	1245.436	1000	1245436	ECOSAR
	fish	2884.581	1000	2884581	ECOSAR
	algae	0.01*	50	200	[9]
IPF	invertebrates	9.06	1000	9060	[10]
	fish	0.001*	50	20	[11]
	algae	176.841	1000	176841	ECOSAR
2-OHIPF	invertebrates	12.804	1000	12804	ECOSAR
	fish	386.193	1000	386193	ECOSAR
	algae	18	1000	18000	[6]
BZB	invertebrates	0.023*	100	230	[12]
	fish	6	1000	6000	[6]
	algae	238.857	1000	238857	ECOSAR
4-CBA	invertebrates	261.503	1000	261503	ECOSAR
	fish	439.828	1000	439828	ECOSAR
	algae	31.6	1000	31600	[1]
CBZ	invertebrates	3.76	1000	3760	[13]
	fish	45.87	1000	45870	[14]

Pharmaceuticals	Aquatic organisms	LC_{50}/EC_{50} (mg/L)	AF	PNEC (ng/L)	Reference
CBZE	algae	463.594	1000	463594	ECOSAR
	invertebrates	326.738	1000	326738	ECOSAR
	fish	61.604	1000	61604	ECOSAR
FLX	algae	0.024	1000	24	[15]
	invertebrates	0.234	1000	234	[15]
	fish	0.546	1000	546	[15]
NFLX	algae	0.160	1000	160	ECOSAR
	invertebrates	0.321	1000	321	ECOSAR
	fish	2.089	1000	2089	ECOSAR
SER	algae	0.457	1000	457	[15]
	invertebrates	0.0048*	100	48	[15]
	fish	0.05	1000	50	[15]
NDS	algae	0.056	1000	56	ECOSAR
	invertebrates	0.130	1000	130	ECOSAR
	fish	0.787	1000	787	ECOSAR
VFS	algae	129.975	1000	129975	ECOSAR
	invertebrates	150.494	1000	150494	ECOSAR
	fish	265.634	1000	265634	ECOSAR
OVM-VFS	algae	1.544	1000	1544	ECOSAR
	invertebrates	2.208	1000	2208	ECOSAR
	fish	17.036	1000	17036	ECOSAR
ATP	algae	1.346	1000	1346	ECOSAR
	invertebrates	36.797	1000	36797	ECOSAR
	fish	5.781	1000	5781	ECOSAR
4-OHATP	algae	2.781	1000	2781	ECOSAR
	invertebrates	102.934	1000	102934	ECOSAR
	fish	13.028	1000	13028	ECOSAR

Note: * Data are NOEC (mg/L); ECOSAR: Ecotoxicological structure-activity relationship model.

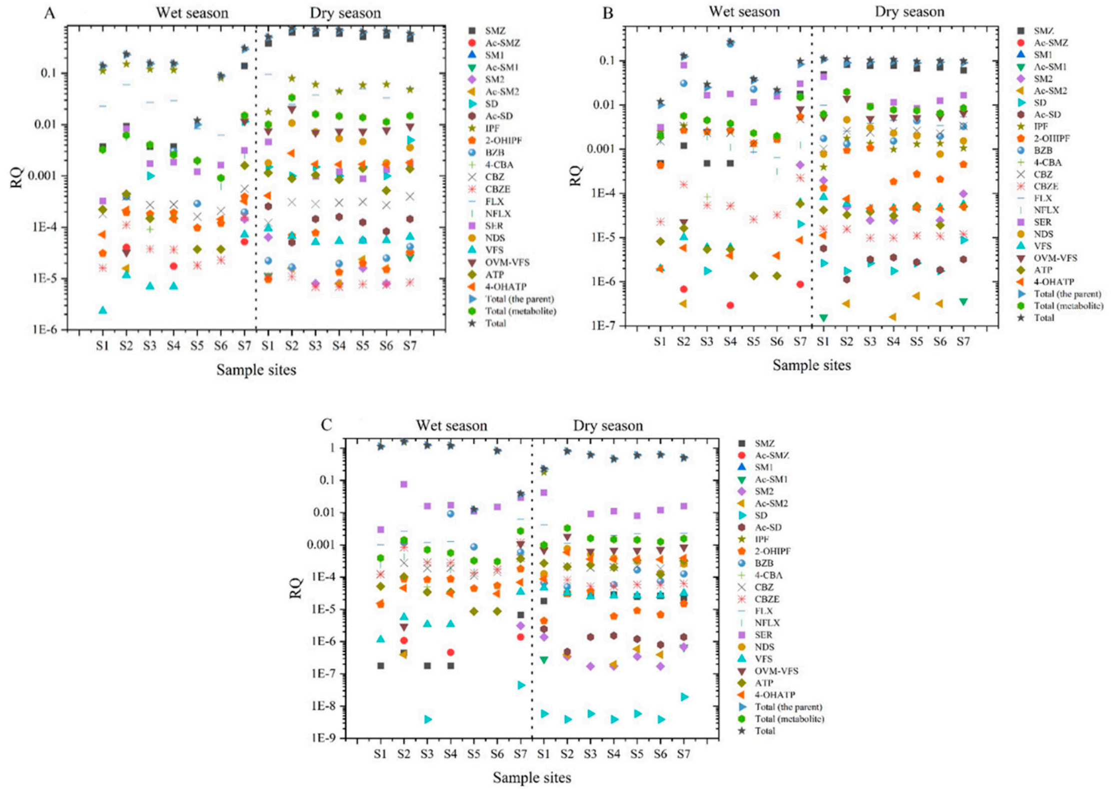

Figure S1. The risks of pharmaceutical metabolites and their parents to algae (A), invertebrates (B), and fish (C) in different sampling sites.

Reference

[1] Ferrari B, Mons R, Vollat B, et al. Environmental risk assessment of six human pharmaceuticals: are the current environmental risk assessment procedures sufficient for the protection of the aquatic environment? Environmental Toxicology and Chemistry, 2004, 23(5): 1344-1354.

[2] Marina I, Margherita L, Angela N, et al. Toxic and genotoxic evaluation of six antibiotics on non-target organisms. The Science of the Total Environment, 2005, 346(1/3): 87-98.

[3] Kim Y, Choi K, Jung J, et al. Aquatic toxicity of acetaminophen, carbamazepine, cimetidine, diltiazem and six major sulfonamides, and their potential ecological risks in Korea. Environment International, 2007, 33(3): 370-375.

[4] Brain R A, Johnson D J, Richards S M, et al. Effects of 25 pharmaceutical compounds to *Lemna gibba* using a seven-day static-renewal test. Environmental Toxicology & Chemistry, 2004, 23(2): 371-382.

[5] Liguoro M D, Fioretto B, Poltronieri C, et al. The toxicity of sulfamethazine to *Daphnia magna* and its additivity to other veterinary sulfonamides and trimethoprim. Chemosphere, 2009, 75(11): 1519-1524.

[6] Kuzmanovic M, Ginebreda A, Petrovic M, et al. Risk assessment based prioritization of 200 organic micropollutants in 4 Iberian rivers. Science of the Total Environment, 2015, 503-504(15): 289-299.

[7] Orte M D, Carballeira C, Viana I G, et al. Assessing the toxicity of chemical compounds associated with marine land-based fish farms: The use of mini-scale microalgal toxicity tests.

Chemistry & Ecology, 2013, 29(6): 554-563.

[8] Li N, Zhang X, Wu W, et al. Occurrence, seasonal variation and risk assessment of antibiotics in the reservoirs in North China. Chemosphere, 2014, 111: 327-335.

[9] Brun G L, Bernier M, Losier R, et al. Pharmaceutically active compounds in atlantic canadian sewage treatment plant effluents and receiving waters, and potential for environmental effects as measured by acute and chronic aquatic toxicity. Environmental Toxicology and Chemistry, 2006, 25(8): 2163-2176.

[10] Pounds N, Maclean S, Webley M, et al. Acute and chronic effects of ibuprofen in the mollusc *Planorbis carinatus* (Gastropoda: Planorbidae). Ecotoxicology and Environmental Safety, 2008, 70(1): 47-52.

[11] Han S, Choi K, Kim J, et al. Endocrine disruption and consequences of chronic exposure to ibuprofen in Japanese medaka (*Oryzias latipes*) and freshwater cladocerans Daphnia magna and Moina macrocopa. Aquatic Toxicology, 2010, 98(3): 256-264.

[12] ISidori M, Nardelli A, Pascarella L, et al. Toxic and genotoxic impact of fibrates and their photoproducts on non-target organisms. Environment International, 2007, 33(5): 635-641.

[13] Quinn B, Gagné F, blaise C. The effects of pharmaceuticals on the regeneration of the cnidarian, *Hydra attenuata*. Science of the Total Environment, 2008, 402(1): 62-69.

[14] Kim J W, Ishibashi H, Yamauchi R, et al. Acute toxicity of pharmaceutical and personal care products on freshwater crustacean (*Thamnocephalus platyurus*) and fish (Oryzias latipes). The Journal of Toxicological Sciences, 2009, 34(2): 227.

[15] Silva L J G, Pereira A M P T, Meisel L M, et al. Reviewing the serotonin reuptake inhibitors (SSRIs) footprint in the aquatic biota: uptake, bioaccumulation and ecotoxicology. Environmental Pollution, 2015, 197: 127-143.
